# LPS-Treated Podocytes Polarize Naive CD4^+^ T Cells into Th17 and Treg Cells

**DOI:** 10.1155/2020/8587923

**Published:** 2020-05-17

**Authors:** Da-Hai Yuan, Yang Jia, Omar Mohamud Hassan, Li-Yun Xu, Xiao-Chuan Wu

**Affiliations:** Department of Pediatric Nephrology, Children's Medical Center, The Second Xiangya Hospital, Central South University, No. 139 Middle Renmin Road, Changsha, Hunan 410011, China

## Abstract

**Aim:**

Our study is aimed at investigating whether Lipopolysaccharide- (LPS-) treated podocytes could polarize naive CD4^+^ T cells into different subsets in vitro.

**Materials and Methods:**

Podocytes and mouse bone marrow-derived dendritic cells (BMDCs) were first cultured with 25 *μ*g/ml LPS for 6 hours, respectively. Then, naive CD4^+^ T cells were cocultured with the LPS-treated podocytes or BMDCs at a ratio of 1 : 1 or 1 : 1 : 1. After 48 hours, we collected the suspended cells and supernatant from all groups to measure T helper (Th)17 cells, regulatory T (Treg) cells, and cytokine concentration.

**Results:**

We observed the expression of CD80 and major histocompatibility complex class II molecule (MHC II) in podocytes but did not found the upregulation of them after treating podocytes with LPS. LPS-treated podocytes could induce naive CD4^+^ T cells to Th17 cells and Treg cells with a higher ratio of Th17/Treg than BMDCs. Possible interaction between podocytes and BMDCs may exist in the induction process of Th17 cells and Treg cells.

**Conclusion:**

Our study proved that CD80 and MHC II were constitutively expressed in podocytes but not upregulated by LPS. LPS-treated podocytes could polarize naive CD4^+^ T cells into Th17 and Treg cells and affect the Th17/Treg balance and may incline to cause a Th17 response.

## 1. Introduction

The exact pathogenesis of idiopathic nephrotic syndrome (INS) remains unclear despite notable progress in our understanding of renal biology. Shalhoub [1] first proposed that the abnormality of T cells was involved in the onset of minimal change disease (MCD). Systemic circulating factors and genetic abnormalities of the podocytes are also related to the pathogenesis of INS [2–5]. The highly differentiated renal podocytes play a vital role in glomerular filtration. Scholars thought INS to be a podocytopathy due to functional and structural changes of the podocytes, leading to massive proteinuria [6]. Moreover, a research suggested that podocytes may have a direct role in the immune disorders of INS [7].

Differentiation of naive CD4^+^ T cells could produce T helper (Th)1, Th2, Th17, and regulatory T (Treg) cells [8]. Scholars have reported Th1/Th2 imbalance and Th17/Treg imbalance existing in INS [9–12]. Th17 cells characteristically express interleukin 17 (IL-17) and transcription factor retinoid-related orphan receptor gamma t (ROR*γ*t) [13, 14]. Plenty of inflammatory and autoimmune diseases involve Th17 cells, including kidney diseases [15]. Some studies have reported IL-17 participates in podocyte damage [16, 17]. Treg cells, which have anti-inflammatory and immune tolerance roles, specifically express forkhead box P3 (Foxp3) [18]. An increased number of Th17 cells and a decreased number of Treg cells were observed in the peripheral blood of primary nephrotic syndrome (PNS) patients [11, 12]. Shao et al. [11] found a dynamic balance of Th17 and Treg cells in children with PNS and suggested that the imbalance of Th17/Treg was involved in the onset and development of PNS.

Podocytes exhibit many characteristics of immune cells [19]. Moreover, podocytes can present antigens and activate CD4^+^ T cells and CD8^+^ T cells, suggests that podocytes could play a role like nonhematopoietic antigen-presenting cells (APCs) [7]. Naive Th cells can differentiate into different subsets mediated by pathogen-primed dendritic cells (DCs) [20], which are the most potent professional APCs. Lipopolysaccharide (LPS), the Toll-like receptor 4 (TLR4) ligand, has diverse effects on DC-mediated T cell polarization. LPS induces Th1 cell polarizing factor production by most mouse and human CD11^+^ DC subsets, so LPS-treated DCs usually support the expansion of Th1 cells [20]. Besides, DCs treated with a low dosage of LPS can induce Th2 immunity, while DCs treated with a high dosage of LPS was followed by a decrease of Th2 immunity and induction of Th17 response [21]. It has also been shown that LPS-treated DCs correlated with the Treg expansion at the DC-draining lymph nodes [22]. Zhou et al. [23] reported that LPS treatment upregulated the expression of tolerance-related factors such as galectin-1 and CD205 on bone marrow-derived DCs (BMDCs) in vitro and suggested that LPS may regulate BMDC-mediated immune tolerance. Given that podocytes are likely to be considered APCs, it is unclear whether podocytes could mediate the polarization of Th cells like DCs and affect the balance of Th cell subsets. So, we used a cell coculture method to study whether LPS-treated podocytes could mediate the polarization of Th cells, aiming to find a new potential therapeutic strategy for INS.

## 2. Materials and Methods

### 2.1. Cell Culture and Treatment

Conditionally immortalized mouse podocyte cell line (PCL) transfected by stable and sensitive SV40T was purchased from Zhong Qiao Xin Zhou Biotechnology, China. PCL cells were cultured in complete medium (RPMI-1640 with 100 U/ml IFN-*γ* and 10% FBS) at 33°C under 5% CO_2_ with the change of the medium every 2 or 3 days, subculturing when the cell confluency reached 70%-80% and for differentiation at 37°C under 5% CO_2_.

C57BL/6 mice were reared in a constant temperature and humidity environment with an artificial light cycle and unrestricted access to standard water and diet. C57BL/6 mice (male, 6-8 weeks) were sacrificed by cervical dislocation for mouse naive CD4^+^ T cells. Mononucleocytes from mouse spleens were collected, and then naive CD4^+^ T cells were isolated with MagniSort™ Mouse CD4 Naive T cell Enrichment Kit (Invitrogen, USA) following the manufacturer's protocol. The percentage of CD4^+^CD45^+^CD44^−^ cells calculated by flow cytometry was 92.51 ± 0.18% (see Figure S1a and S1c in Supplementary Material).

To obtain mouse BMDCs, we sacrificed C57BL/6 mice (male, 6-8 weeks) by cervical dislocation. Collected mononucleocytes from mouse bone marrow and then suspended the cells in RPMI-1640 medium with antibiotics and 10% FBS. Mouse IL-4 (Abcam, UK) and granulocyte-macrophage colony-stimulating factor (GM-CSF) (Abcam, UK) were added into the medium to a concentration of 10 ng/ml and 20 ng/ml, respectively [24]. The medium was changed on day 3. Floating or lightly adherent cells were collected on day 7. Then, we further purified the collected cells with CD11c MicroBeads (Miltenyi, Germany) following the manufacturer's protocol. So BMDCs were harvested. The percentage of CD11c^+^ cells calculated by flow cytometry was 97.66 ± 0.21% (see Figure S1a and S1c in Supplementary Material).

In a previous study, LPS (25 *μ*g/ml) induced an increase of TLR 1–4 gene expression in podocytes within 1 hour, and the expression of TLR4 had begun to decline by 6 hours [25]. Herein, in our study, PCL cells were cultured with 25 *μ*g/ml LPS (eBioscience, USA) for 6 hours, and the harvested BMDCs were cultured with 25 *μ*g/ml LPS for 6 hours, too. Before coculture, we examined the levels of CD80, major histocompatibility complex class II molecule (MHC II), and TLR4 mRNA expressed in PCL cells and BMDCs.

### 2.2. Coculture

After 6 hours of treatment with LPS, PCL cells and BMDCs were washed by PBS. 2 × 10^5^ naive CD4^+^ T cells were cocultured with LPS-treated PCL cells or BMDCs at a ratio of 1 : 1 or 1 : 1 : 1. One control group (naive CD4^+^ T cells only) and three coculture groups (BMDCs+naive CD4^+^ T cells, PCL cells+naive CD4^+^ T cells, and PCL cells+BMDCs+naive CD4^+^ T cells) were established. After 48 hours of culture at 37°C under 5% CO_2_, suspended cells and supernatant in each group were collected for further study.

### 2.3. Flow Cytometry

For flow cytometry, we used monoclonal antibodies CD11c-FITC, CD80-PE, MHC II-APC, CD4-PE-Cyanine7, CD44-PE, CD45-FITC, Foxp3-PE, CD25-APC, and IL-17A-APC (eBioscience, USA). Cells were incubated with monoclonal antibodies for 30 min in the dark for the staining of surface antigens. IL-17A and Foxp3 were performed by intracellular staining.

### 2.4. Quantitative Real-Time Polymerase Chain Reaction (qRT-PCR)

For investigating the TLR4 mRNA expression in PCL cells and BMDCs after LPS treatment, we performed qRT-PCR. TRIzol Reagent (Invitrogen, USA) was used to extract total RNA following the manufacturer's protocol. Reverse transcribing total RNA using HiFiScript cDNA Synthesis Kit (Cwbiotech, China). UltraSYBR Mixture (Cwbiotech, China) was used for the qRT-PCR reaction. The reaction condition was as follows: heating to 95°C for 10 min, 40 cycles of 95°C for 15 sec, and 60°C for 30 sec. The relative expression levels of TLR4 mRNA were evaluated by the 2^-*ΔΔ*Cq^ method. The internal control was performed by actin mRNA. Primer Premier 5.0 software (Premier, CAN) was used to design the specific primer. The primer sequences of TLR4 were as follows: forward primer, AGACACTTTATTCAGAGCCGTTG; reverse primer, AAGGCGATACAATTCCACC. The primer sequences of actin were as follows: forward primer, ACATCCGTAAAGACCTCTATGCC; reverse primer, TACTCCTGCTTGCTGATCCAC.

### 2.5. Enzyme-Linked Immunosorbent Assay (ELISA)

To detect the concentration of interferon-gamma (IFN-*γ*), IL-4, and IL-17A in the supernatant of each group, we used colorimetric sandwich ELISA kits (Proteintech, USA) following the manufacturer's instructions.

### 2.6. Statistical Analysis

Data are all shown as the mean ± SEM. Statistical analysis and data graphics were performed using SPSS statistics 25.0 software (IBM, USA) and GraphPad Prism 8 (GraphPad Software Inc., USA). One-way analysis of variance (ANOVA) was used for multiple comparisons. *P* < 0.05 was considered to be statistically significant.

## 3. Results

### 3.1. The Expression of CD80, MHC II, and TLR4 mRNA in LPS-Treated Podocytes and BMDCs

**Figure 1:**
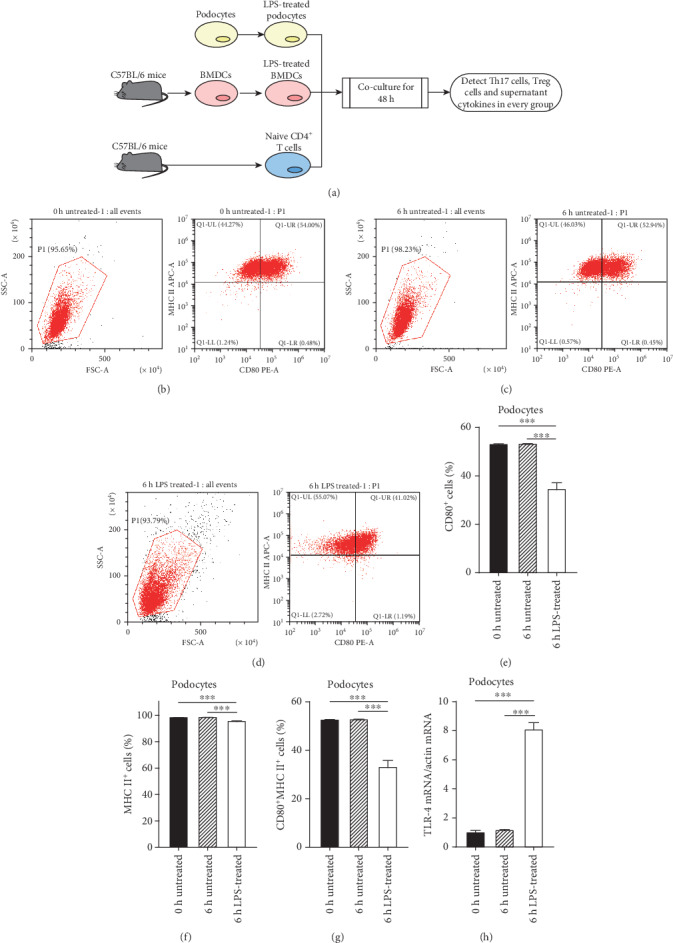
The expression of CD80, MHC II, and TLR4 mRNA in LPS-treated podocytes. PCL cells were cultured for 6 hours with or without 25 *μ*g/ml LPS. CD80, MHC II, and TLR4 mRNA were examined and compared among the 0 h untreated, 6 h untreated, and 6 h LPS-treated groups. CD80 and MHC II were measured by flow cytometry while TLR4 mRNA by qRT-PCR. (a) Schematic diagram of this study. (b–d) The levels of CD80 and MHC II expressed in podocytes in the 0 h untreated group (b), 6 h untreated group (c), and 6 h LPS-treated group (d) were calculated, respectively. (e–h) Comparison of the CD80, MHC II, and TLR4 mRNA expressions in podocytes among groups. Data are shown as the mean ± SEM. ^∗∗∗^*P* < 0.001. *n* = 5/group. PCL: podocyte cell line; BMDCs: bone marrow-derived dendritic cells.

Figure 1(a) shows a schematic diagram of this study.

The untreated groups showed PCL cells constitutively express CD80 and MHC II (Figures 1(b), 1(c), 1(e), 1(f), and 1(g)). After treatment with 25 *μ*g/ml LPS, the levels of CD80 and MHC II in PCL cells were not upregulated but downregulated compared to the untreated groups (Figures 1(b)–1(g)). As expected, the upregulation of TLR4 mRNA was observed in LPS-treated PCL cells (Figure 1(h)).

**Figure 2:**
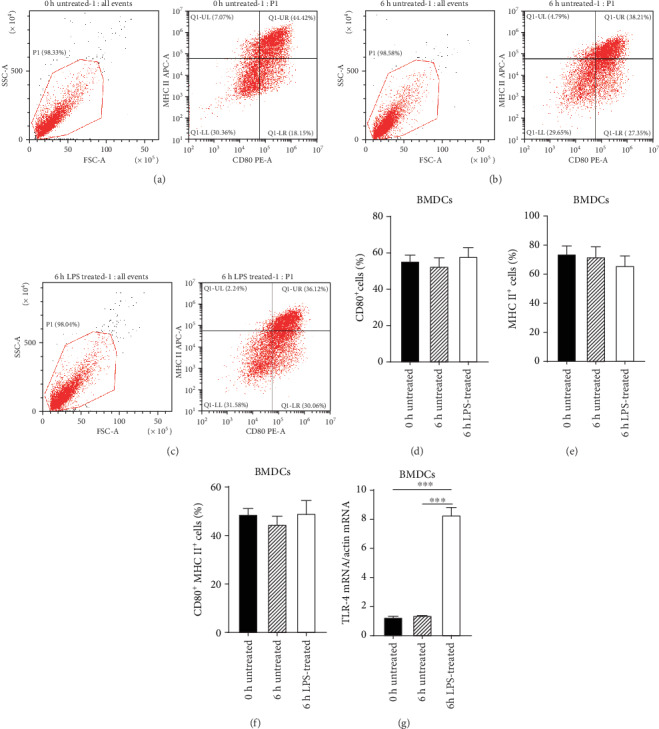
The expression of CD80, MHC II, and TLR4 mRNA in LPS-treated BMDCs. BMDCs obtained from C57BL/6 mice were cultured for 6 hours with or without 25 *μ*g/ml LPS. CD80, MHC II, and TLR4 mRNA were examined and compared among the 0 h untreated, 6 h untreated, and 6 h LPS-treated groups. CD80 and MHC II were measured by flow cytometry while TLR4 mRNA by qRT-PCR. (a–c) The levels of CD80 and MHC II expressed in BMDCs in the 0 h untreated group (a), 6 h untreated group (b), and 6 h LPS-treated group (c) were calculated, respectively. (d–g) Comparison of CD80, MHC II, and TLR4 mRNA expressions in BMDCs among groups. Data are shown as the mean ± SEM. ^∗∗∗^*P* < 0.001. *n* = 5/group. BMDCs: bone marrow-derived dendritic cells.

After treatment with LPS, the levels of CD80 and MHC II expressed in BMDCs were not significantly different from the untreated groups (Figures 2(a)–2(f)), indicating that the harvested BMDCs were mature enough. There was an increase in TLR4 mRNA observed in LPS-treated BMDCs (Figure 2(g)).

### 3.2. LPS-Treated Podocytes Could Induce Naive CD4^+^ T Cells to Th17 Cells and Treg Cells

**Figure 3:**
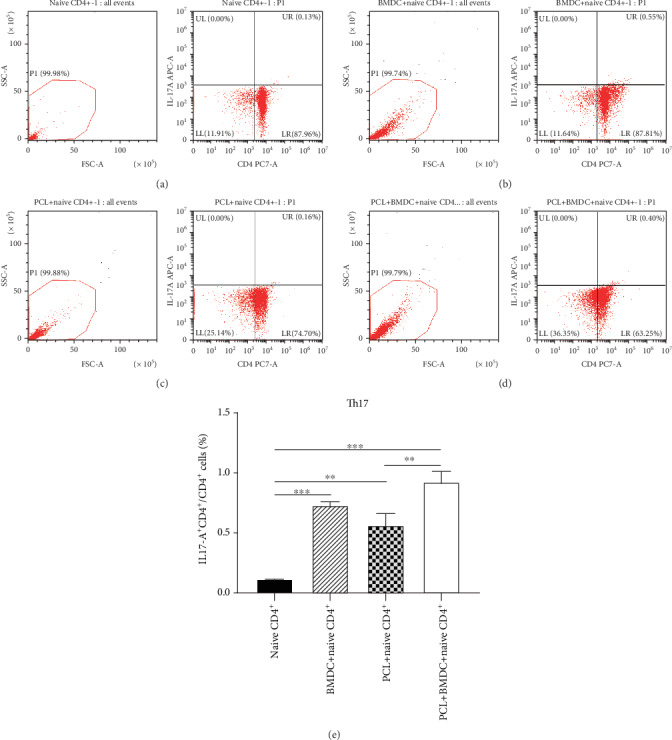
LPS-treated podocytes could induce naive CD4^+^ T cells to Th17 cells (CD4^+^IL-17A^+^ cells). LPS-treated PCL cells or BMDCs were cocultured with naive CD4^+^ T cells (obtained by magnetic cell sorting) at a ratio of 1 : 1 or 1 : 1 : 1. One control group (naive CD4^+^ T cells only) and three coculture groups (BMDCs+naive CD4^+^ T cells, PCL cells+naive CD4^+^ T cells, and PCL cells+BMDCs+naive CD4^+^ T cells) were established. After 48 hours, suspended cells in each group were collected for further study. (a–d) The levels of Th17 cells in the naive CD4^+^ T cell group (a), BMDC+naive CD4^+^ T cell group (b), PCL cell+naive CD4^+^ T cell group (c), and PCL cell+BMDC+naive CD4^+^ T cell group (d) were calculated by flow cytometry, respectively. (e) Comparison of the levels of Th17 cells in total CD4^+^ T cells among groups. Data are shown as the mean ± SEM. ^∗∗^*P* < 0.01. ^∗∗∗^*P* < 0.001. *n* = 5/group. BMDC: bone marrow-derived dendritic cell; PCL: podocyte cell line.

In the three coculture groups, the levels of Th17 cells (CD4^+^IL-17A^+^ cells) in total CD4^+^ cells elevated significantly compared to the control group (Figures 3(a)–3(e)), indicating that LPS-treated podocytes and BMDCs both can induce naive CD4^+^ T cells to Th17 cells. Moreover, in the PCL cell+BMDC+naive CD4^+^ T cell group, the elevated Th17 cells was higher than the PCL cell+naive CD4^+^ T cell group (Figure 3(e)).

**Figure 4:**
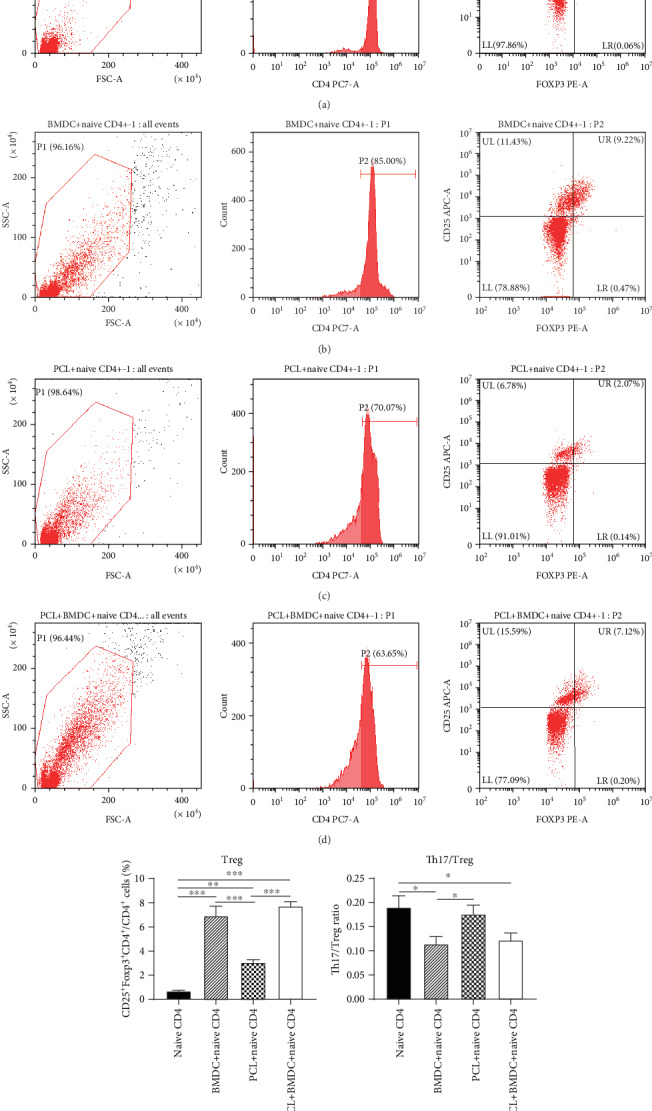
LPS-treated podocytes induce naive CD4^+^ T cells to Treg cells (CD4^+^CD25^+^Foxp3^+^ cells). After 48 hours of coculture, suspended cells in each group were collected for further study. (a–d) The levels of Treg cells in the naive CD4^+^ T group (a), BMDC+naive CD4^+^ T cell group (b), PCL cell+naive CD4^+^ T cell group (c), and PCL cell+BMDC+naive CD4^+^ T cell group (d) were calculated by flow cytometry, respectively. (e) Comparison of the levels of Treg cells in total CD4^+^ T cells among groups. (f) Comparison of the Th17/Treg ratio among culture groups. Data are shown as the mean ± SEM. ^∗^*P* < 0.05. ^∗∗^*P* < 0.01. ^∗∗∗^*P* < 0.001. *n* = 5/group. BMDC: bone marrow-derived dendritic cell; PCL: podocyte cell line.

In the three coculture groups, the levels of Treg cells (CD4^+^CD25^+^Foxp3^+^ cells) in total CD4^+^ cells raised significantly compared to the control group (Figures 4(a)–4(e)), indicating that LPS-treated podocytes and BMDCs both can induce naive CD4^+^ T cells to Treg cells. The raised Treg cells in the PCL cell+naive CD4^+^ T cell group was lower than the BMDC+naive CD4^+^ T cell group (Figure 4(e)). Moreover, in the PCL cell+BMDC+naive CD4^+^ T cell group, the elevated level of Treg cells had a more significant range than that of the PCL cell+naive CD4^+^ T cell group (Figure 4(e)).

Together, these findings proved that LPS-treated podocytes could induce naive CD4^+^ T cells to Th17 cells and Treg cells. Podocytes have a less powerful role than BMDCs in promoting the production of Treg cells from naive CD4^+^ T cells. Podocytes and BMDCs cultured together had a more robust role than podocytes alone in the induction process of Th17 cells and Treg cells.

### 3.3. LPS-Treated Podocytes Induced a Higher Th17/Treg Ratio Than BMDCs

To evaluate the effect of LPS-treated podocytes on Th17/Treg balance, we further compared the ratio of Th17/Treg among coculture groups (Figure 4(f)). The PCL cell+naive CD4^+^ T cell group had a higher Th17/Treg ratio than the BMDC+naive CD4^+^ T cell group (Figure 4(f)).

### 3.4. Supernatant Cytokine Concentration in Coculture Groups

**Figure 5:**
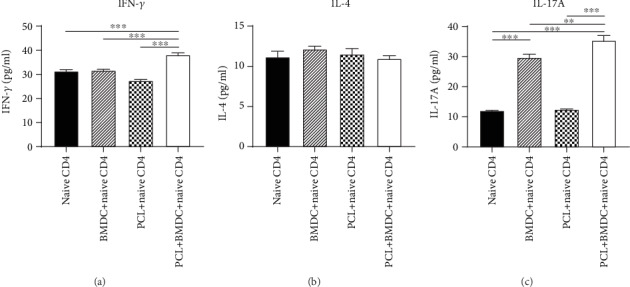
The concentrations of supernatant cytokines in coculture groups. After 48 hours of coculture, the supernatant in each group was collected for further study. IFN-*γ* (a), IL-4 (b), and IL-17A (c) in each group were examined by ELISA. Data are shown as the mean ± SEM. ^∗∗^*P* < 0.01. ^∗∗∗^*P* < 0.001. *n* = 5/group. BMDC: bone marrow-derived dendritic cell; PCL: podocyte cell line.

The concentration of Th1 cytokine IFN-*γ* in the PCL cell+naive CD4^+^ T cell and BMDC+naive CD4^+^ T cell groups was not elevated compared to the control group (Figure 5(a)). However, the concentration of IFN-*γ* in the PCL cell+BMDC+naive CD4^+^ T cell group was significantly higher than those in the other three groups (Figure 5(a)).

The concentrations of Th2 cytokine IL-4 in all three coculture groups were not significantly elevated (Figure 5(b)).

IL-17A secretion was detectable in the BMDC+naive CD4^+^ T cell group but not in the PCL cell+naive CD4^+^ T cell group (Figure 5(c)). Moreover, the concentration of IL-17A in the PCL cell+BMDC+naive CD4^+^ T cell group was significantly higher than those in the other three groups (Figure 5(c)).

## 4. Discussion

The reported incidence of INS in children is 1.15 to 16.9/100000 [6]. At present, the main treatment agents for INS are glucocorticoids and other immunosuppressants such as tacrolimus and mycophenolate mofetil. Nevertheless, drug side effects and high recurrence rates have constantly been clinical problems. Therefore, new treatment strategies are urgently needed. Since Shalhoub [1] proposed that MCD was a disorder of T cells which disrupted the glomerular basement membrane by producing a circulating factor, it has been considered that the pathogenesis of INS involves T cell dysfunction for decades. However, new shreds of evidence suggest that podocytes have a direct role in the occurrence and recurrence of INS.

Podocytes share many factors' characteristics (including CD80 and MHC II) of the immune system and are likely to be considered APCs [7, 19]. There was no direct evidence shown that CD80 and MHC II could indicate the activation or maturation of PCL cells, but the expression of CD80 and MHC II on PCL cells could exhibit some of the features of APCs [7, 26]. CD80 (also known as B7-1), essential for the activation of T cells, is a costimulatory molecule expressed on the surface of APCs. Increased expression of CD80 in podocytes may be involved in the pathogenesis of proteinuria by breaking the glomerular filter [26, 27]. The CTLA-4 mimicking therapeutic agents, which binds CD80, have been tested in CD80-positive proteinuric kidney disease [28]. Our previous study proved that the change of urinary CD80 concentration could predict the recurrence of steroid-sensitive nephrotic syndrome [29]. Nevertheless, the CD80 hypothesis remains controversial [30, 31]. Reiser et al. [26] reported that LPS treatment upregulated the expression of CD80 in podocytes and led to severe proteinuria in WT and SCID mice but not in CD80^–/–^ mice. In our current study, podocytes constitutively expressed CD80 and MHC II. Upregulations of TLR4 mRNA in podocytes and BMDCs after treatment with LPS proved the effectiveness of LPS. However, there was no upregulation of CD80 in the LPS-treated podocytes (Figure 1(e)), which was consistent with a recent study [31]. So, the CD80 hypothesis requires further confirmation.

APCs include several kinds of cells, such as macrophages, dendritic cells, and B cells. It has been reported that DCs are more efficient at presenting antigens to naive T cells than macrophages [32]. Moreover, the stimulatory effect of macrophages on naive T cells remains controversial. Bartz et al. [33] have shown that DCs had a strong stimulating effect on allogeneic naive T cells, while macrophages had no stimulating effect on those cells. It is considered that B cells are indolent APCs due to their inefficiency in capturing and processing antigens compared to DCs or macrophages [34]. LPS-treated DCs can induce naive Th cells to Th1, Th2, Th17, or Treg cells depending on different conditions [20–22]. Therefore, we added BMDCs rather than other kinds of APCs into the coculture system with naive CD4^+^ T cells as a positive control group. Since podocytes are considered APCs, podocytes may also have a similar role in mediating the polarization of Th cells. A previous study reported podocytes stimulated the secretion of Th1 cytokines IFN-*γ* and IL-2 by CD4^+^ cells [7]. Our current study further proved that LPS-treated podocytes could induce naive CD4^+^ T cells to Th17 cells and Treg cells (Figures 3(e) and 4(e)). The elevated Treg cells in the PCL cell+naive CD4^+^ T cell group were less than those in the BMDC+naive CD4^+^ T cell group (Figure 4(e)), meaning that podocytes have a less influential role than BMDCs in inducing the production of Treg cells from naive CD4^+^ T cells. Th17 cells play crucial roles in the induction of inflammatory and autoimmune diseases, while Treg cells have an anti-inflammatory role [15, 18]. Previous researches suggested that Th17 cells decreased the podocalyxin level and induced podocytes apoptosis [35]. The protective role of Treg cells in INS was supported by animal experiments [36]. So, due to a higher Th17/Treg ratio (Figure 4(f)), LPS-treated podocytes may more incline to cause inflammatory or autoimmune response compared with LPS-treated BMDCs. These results were implying that in addition to being a victim, podocytes also may actively participate in the immune disorders of INS by affecting the balance of Th17/Treg and promoting the local inflammatory response.

As DCs and podocytes may exist at the same microenvironment, they have the opportunity to contact each other. Meanwhile, they both function as APCs, so we hypothesize that there was an interaction between podocytes and DCs in mediating the polarization of Th cells. Therefore, we added BMDCs into a coculture system of PCL cells with naive T cells. The results indicated that podocytes and BMDCs cultured together had a more influential role than podocytes alone in the induction process of Th17 cells and Treg cells (Figures 3(e) and 4(e)), which may be due to the superposition effect or interaction between podocytes and BMDCs. The increase of Th1 cytokine IFN-*γ* in the PCL cell+naive CD4^+^ T cell and BMDC+naive CD4^+^ T cell groups was not observed compared to that in the control group (Figure 5(a)). However, when LPS-treated PCL cells and BMDCs were cultured together with naive CD4^+^ T cells (the PCL cell+BMDC+naive CD4^+^ T cell group), the concentrations of IFN-*γ* was significantly increased (Figure 5(a)), indicating possible interaction between podocytes and BMDCs. The increase of IL-17A in the PCL cell+BMDC+naive CD4^+^ T cell group was observed and significantly higher than that in the BMDC+naive CD4^+^ T cell group (Figure 5(c)), suggesting podocytes and BMDCs cultured together had a more influential role than BMDCs alone in promoting the secretion of IL-17A. Considering IL-17A secretion was undetectable in the PCL cell+naive CD4^+^ T cell group (Figure 5(c)), this may be due to interaction but not the superposition effect between podocytes and BMDCs. Together, it seemed that possible interaction between podocytes and DCs in inducing Th17 cells, Treg cells, IFN-*γ*, and IL-17A existed in our study. However, it needs to be further confirmed by a more excellent experiment. If the interaction does exist, it will provide a new direction for the treatment of INS. There is no research aimed at the aspect by now. So, it is necessary to study the interaction between podocytes and DCs in the future work.

Previous studies reported that LPS-treated DCs usually supported the Th1 response and DCs treated with a low dosage of LPS led to a Th2 response [20, 21]. Moreover, podocytes promoted the secretion of Th1 cytokines IFN-*γ* and IL-2 by CD4^+^ cells [7]. However, in our study, increasing of Th1 cytokine IFN-*γ* and Th2 cytokine IL-4 in the PCL cell+naive CD4^+^ T cell group and BMDC+naive CD4^+^ T cell group was not detectable (Figures 5(a) and 5(b)); it may be due to the high dosage of LPS (25 *μ*g/ml). Peters et al. [21] have reported that BMDCs treated with a high dosage of LPS (100 ng/ml) led to a decrease of Th2 immunity and induction of Th17 response. They found that the secretion of transforming growth factor-beta (TGF-*β*) and IL-6 continuously increased until 100 ng/ml LPS [21]. TGF-*β* and IL-6 are the polarizing factors for the differentiation of Th17 cells [37], and IL-6 could negatively regulate Th1 cell differentiation [38]. This may explain why Th1 cytokine IFN-*γ* and Th2 cytokine IL-4 were not induced.

Compared to the control group, Th17 cell cytokine IL-17A in the PCL cell+naive CD4^+^ T cell group was not increased significantly (Figure 5(c)). However, in the PCL cell+naive CD4^+^ T cell group, the increase of Th17 cells calculated by flow cytometry was observed (Figure 3(e)). The two results were inconsistent. We hypothesized that the intracellular IL-17A might not be entirely secreted outside the cells. The expression of IL-17A was performed by intracellular staining when calculating Th17 cells by flow cytometry, so its upregulation could be detectable. Moreover, Yan et al. [17] have reported that mouse podocytes expressed IL-17A receptor continuously and IL-17A bound to the IL-17A receptor further caused podocytes injury through the ROS-NLRP3-inflammasome-caspase-1 pathway. It could support our hypothesis that the PCL-induced secretion of IL-17A may be overridden by the binding to the IL-17A receptor on podocytes. This may explain why PCL cells could induce naive CD4^+^ T cells to Th17 cells, but IL-17A secretion was not detected in the supernatant of PCL cell+naive CD4^+^ T cell group.

## 5. Conclusions

In conclusion, our study proved that CD80 and MHC II constitutively expressed in podocytes but not upregulated by LPS. LPS-treated podocytes could polarize naive CD4^+^ T cells into Th17 and Treg cells and affect the Th17/Treg balance and may promote the local inflammatory response. This may provide a new potential treatment strategy for INS. However, in vivo verification and the specific signal pathways need further investigation.

## Data Availability

All data used to support the results of this study are available from the corresponding author upon request.

## Supplementary Materials

Supplementary Materials Figure S1: the identification of naive CD4^+^ T cells and BMDCs.
