# Supplementary material for: LPS-Treated Podocytes Polarize Naive CD4+ T Cells into Th17 and Treg Cells
Source: Biomed Res Int. 2020 May 17;2020:8587923. doi: 10.1155/2020/8587923 (PMC7251438; doi:10.1155/2020/8587923)
Supplement: Supplementary Materials — Figure S1: the identification of naive CD4+ T cells and BMDCs. [file 8587923.f1.pdf]

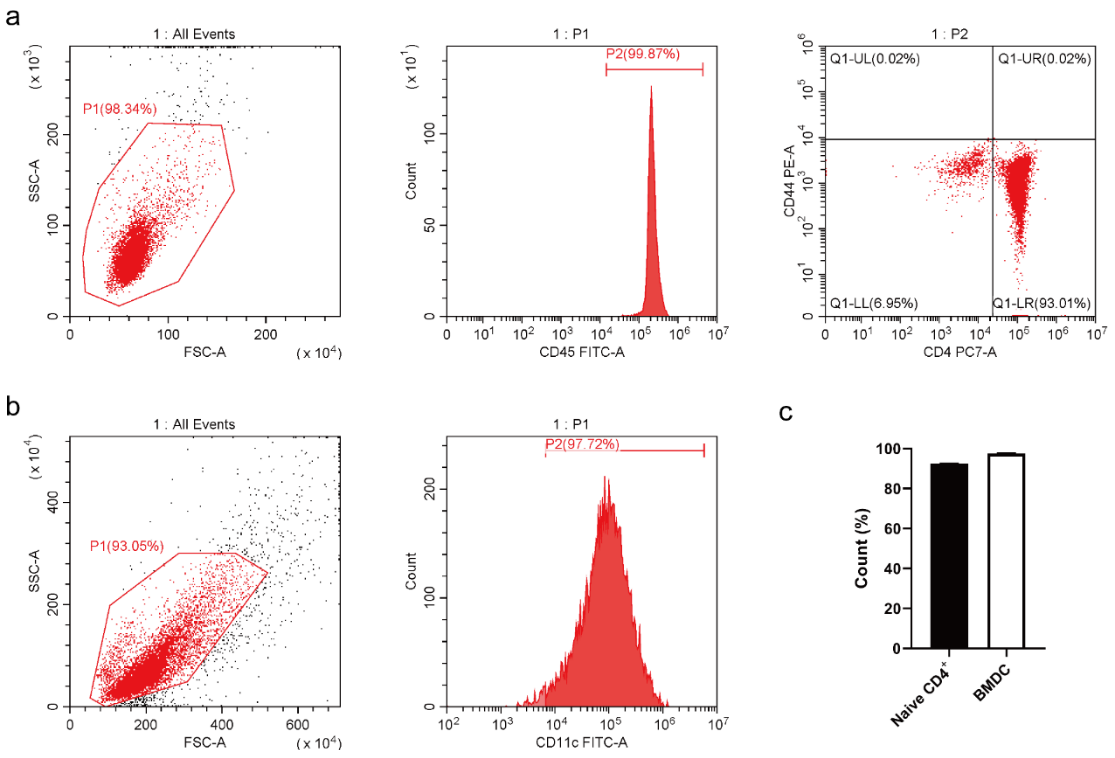

Figure S1 The identification of naive $CD4^+$ T cells and BMDCs.

Naive $CD4^+$ T cells were obtained from C57BL/6 mouse spleens by magnetic cell sorting. BMDCs were induced from bone marrow mononucleocytes of C57BL/6 mice. Flow cytometry was performed for cell identification. (a) The expression of CD45, CD4, and CD44 in obtained naive $CD4^+$ T cells were calculated. (b) The expression of CD11c in induced BMDCs was calculated. (c) The expression levels of $CD4^+CD45^+CD44^-$ naive $CD4^+$ T cells and $CD11c^+$ BMDCs were calculated. (Data are shown as mean ± SEM. BMDC, bone marrow-derived dendritic cell.)
